# Prevalence and Awareness of Diabetic Retinopathy in Diabetic Patients Visiting Tertiary Care Hospitals in Central India

**DOI:** 10.7759/cureus.39414

**Published:** 2023-05-23

**Authors:** Shubhangi Setia, Pravin Tidake

**Affiliations:** 1 Medicine, Jawaharlal Nehru Medical College, Datta Meghe Institute of Higher Education and Research, Wardha, IND; 2 Ophthalmology, Jawaharlal Nehru Medical College, Datta Meghe Institute of Higher Education and Research, Wardha, IND

**Keywords:** hyperglycemia, npdr, pdr, etdrs, retinopathy, diabetes

## Abstract

Background

Diabetes presents a significant and ever-growing burden worldwide, impacting populations across the globe. However, this burden is particularly pronounced in developing economies like India. The rise of diabetes in such regions can largely be attributed to two key factors: the increasing prevalence of overweight/obesity and the adoption of unhealthy lifestyles.

Aim and objectives

The study's objectives encompassed identifying patients with type 2 diabetes mellitus through the oral glucose tolerance test and the diagnosis of diabetic retinopathy (DR) among them, followed by classification based on the severity of the disease. Furthermore, the study aimed to evaluate the factors associated with DR among the patients, focusing on the duration of diabetes and age group, both of which were deemed highly significant.

Methods

This cross-sectional study was conducted for two months in 2019 among 40 patients. The tests performed were an oral glucose tolerance test, ocular examination including slit lamp biomicroscopy, and fundus examination. The data were analyzed using the Chi-square test and Student T-test.

Results

About 42.5% of the patients were positive for diabetic retinopathy, of which 29.41% had mild NPDR, 41.18% had moderate non-proliferative diabetic retinopathy (NPDR), and 29.41% had diabetic maculopathy. No cases of severe NPDR and proliferative diabetic retinopathy (PDR) were found.

Conclusion

Longer duration of diabetes corresponded to a higher prevalence of diabetic retinopathy. The frequency of diabetic retinopathy was higher in ages above 60 years. Diabetic retinopathy is a concern for patients with a long history of diabetes, high blood sugar levels, and unilateral or bilateral low vision. Thirty percent of the total were newly diagnosed diabetics. Timely screening of patients and intervention can go a long way in reducing morbidity or vision loss.

## Introduction

According to the World Health Organization (WHO), diabetes affects a significant portion of the global adult population. In 2014, the WHO estimated that approximately 8.5% of adults worldwide are affected by diabetes [1]. Diabetes is a chronic metabolic disorder characterized by elevated blood sugar levels, and it can lead to various complications if not properly managed. According to the Indian Council of Medical Research, diabetes mellitus affects approximately 8.4% of the population in Maharashtra, India [2]. This indicates a relatively high prevalence of diabetes in this region. The factors contributing to this high prevalence may include urbanization, sedentary lifestyles, unhealthy dietary habits, genetic predisposition, and a lack of awareness about diabetes management.

Diabetic retinopathy is a condition that arises as a complication of diabetes. It involves irreversible damage to the microvascular system in the retina, which is the light-sensitive tissue at the back of the eye. Prolonged elevated blood sugar levels contribute to the development and progression of this condition. Additionally, diabetic retinopathy is associated with various hematological (related to blood) and biochemical changes [1-2].

The statement also highlights a lack of research regarding the prevalence of diabetic retinopathy, specifically in the Vidarbha region. Vidarbha is a region located in the state of Maharashtra, India. The absence of sufficient studies in this area emphasizes the critical need for further research to determine the extent of diabetic retinopathy and its impact on the population in Vidarbha. Raising awareness about diabetic retinopathy is essential to ensure that affected individuals receive the appropriate care and attention. Early detection and timely treatment are crucial for managing diabetic retinopathy and preventing vision loss. By increasing awareness, healthcare professionals, community organizations, and individuals can work together to identify and support those with diabetic retinopathy, promoting better eye health and overall well-being.

## Materials and methods

Study setting and design

This study was a cross-sectional study conducted at the Department of Ophthalmology in a 250-bed tertiary care hospital in central India. The study spanned two months, specifically from July 1, 2019, to August 31, 2019. Approval for the study was obtained from the Institutional Ethical Committee. A total of 40 patients, who were diagnosed with diabetes mellitus (DM) and were attending the Medicine Outpatient Department (OPD), were selected for the study. Additionally, diabetic patients visiting the Ophthalmology OPD were also included. Before participation, the patients were provided with a translated explanation of the study's purpose, implications, and methodology in their local language and obtained their written consent.

Sample size calculation

The sample size calculation for this study was conducted using high-standard American English. The confidence interval (z) was set at 1.96, indicating the desired confidence level. The error of margin (d) was determined to be 7%, representing the acceptable error range in the results. The prevalence of DR (p) was established as 5.46%, based on reference sources such as the prevalence of DR in 65% of Maharashtra patients and 8.4% according to the Indian Council of Medical Research (ICMR). By plugging these values into the formula, the sample size (n) was computed as 40. The resulting sample size of 40 was considered sufficient for the study's objectives.

Sample characteristics (inclusion and exclusion criteria)

The study included patients between the ages of 18 and 80 who were diagnosed with type 2 diabetes mellitus and provided their consent. However, patients with mature cataracts and hazy media, hypertensive retinopathy, sickle cell disease, known radiation exposure, secondary diabetes, those taking medications that could affect blood glucose levels, and patients with type 1 diabetes mellitus were excluded from the study. These exclusions were made because these conditions or factors could potentially lead to false positive findings for diabetic retinopathy. Additionally, patients who did not provide consent were also excluded.

Data collection process and method

Data were collected for a total of 40 individuals who were diagnosed with diabetes. This group consisted of individuals with pre-existing cases and their respective durations and newly diagnosed cases. The diagnosis of diabetes mellitus (DM) encompassed three distinct possibilities. The first category consisted of individuals already known to have diabetes and were treated with either oral anti-diabetic drugs, insulin, or a combination of both. For these individuals, the duration of diabetes was assigned a value of zero. The second category encompassed individuals whose fasting blood glucose levels, as determined by the capillary method, exceeded 100mg/dl. These individuals underwent an oral glucose tolerance test (OGTT). A diagnosis of newly diagnosed diabetes was made when the OGTT yielded a result of 200mg/dl or higher. The third and final category was sight-threatening diabetic retinopathy (STDR), which included clinically significant macular edema (CSME), diabetic macular edema (DMO), severe non-proliferative diabetic retinopathy (NPDR), and proliferative diabetic retinopathy (PDR).

Subsequently, a proforma was completed, outlining pertinent clinical history. This comprehensive record encompassed patient demographics, assessment of presenting complaints, and concurrent conditions such as hypertension or cataract. The most prevalent indicators and symptoms comprised floaters, blurred vision, fluctuating vision, impaired color vision, dark or empty areas in vision, and complete vision loss. A thorough examination of the patients was conducted, and their vital signs were recorded. This encompassed pulse, blood pressure, peripheral pulses, higher functions evaluation, and higher cranial functions assessment. Additionally, an examination of the ears, nose, and throat, measurement of blood sugar levels, determination of glycated hemoglobin A1c (HbA1c) concentration, and evaluation of insulin usage (if applicable) were also performed.

Subsequently, they underwent a comprehensive ocular examination to detect diabetic retinopathy (DR). Initially, the visual acuity was evaluated utilizing Snellen's chart. The initial uncorrected visual acuity (UCVA) at a distance was recorded for both eyes using Snellen's charts and/or Illiterate E charts. If the UCVA was below 6/6, any improvement with a pinhole was also documented. This was followed by auto-refractometry. The external examination of the eyes was performed using a flashlight to identify conditions such as exophthalmos and enophthalmos. Any abnormalities in the position of the eyeball were recorded. A standard ocular examination was then conducted. The depth of the anterior chamber was assessed to identify any opacity in the media, such as corneal haziness, evidence of pseudophakia, or aphakia. Detailed observations were made of the anterior segment, encompassing the lids to the lens. Additionally, the anterior vitreous was examined to detect the presence of cells and pigment. Subsequently, the intraocular pressure was measured using a non-contact tonometer or Schiotz tonometer. Three readings were taken and averaged, with the normal range of 10-21 mm of mercury. The eyeballs' movements were carefully documented, noting versions and ductions in all cardinal planes.

A comprehensive examination of the posterior segment was also conducted. In patients with normal intraocular pressure and normal anterior chamber depth observed through the slit lamp, the pupils were dilated using 0.8% tropicamide eye drops containing 5% phenylephrine. Subsequently, fundus examination was performed using a direct, indirect microscope, or slit lamp bio-microscope equipped with a 90 D lens. This examination aimed to assess optic disc changes and diagnose diabetic retinopathy. During the slit lamp examination, a visual acuity test known as best-corrected visual acuity (BCVA) was conducted. If the BCVA in the better eye was equal to or below 0.1, it was classified as legal blindness. BCVA ranging from 0.2 to 0.4 was categorized as low vision. In cases where the BCVA in the better eye was greater than 0.5, and the fellow eye was equal to or below 0.1, it was referred to as unilateral blindness. If the BCVA in the fellow eye ranged from 0.2 to 0.4, it was classified as unilateral low vision. In the event of a successful diagnosis of diabetic retinopathy, the findings were further categorized as non-proliferative DR (NPDR) or proliferative DR (PDR) based on the Modified Early Treatment Diabetic Retinopathy Study Research (ETDRS). This classification system considers the appearance and severity of microaneurysms, retinal hemorrhages, the affected quadrants, the presence of retinal edema, hard exudates, cotton wool spots, venous beading, and intraretinal microvascular abnormalities (IRMA) [3].

Additionally, the patients were asked about their knowledge regarding the long-term complications of diabetes mellitus, including DR, nephropathy, and neuropathy. This inquiry aimed to assess the level of awareness among patients regarding these significant issues. Any information provided by the patients, ranging from general terms like "eye disease" to specific mention of "retinal complications," was duly considered.

Statistical analysis

The data were analyzed utilizing the Chi-square test and Student T-test to compare variables. The software was the Statistical Package for Social Sciences (SPSS) 17.0 (IBM Corporation, Armonk, New York, United States) and Graph Pad 6.0.

Ethical consideration

Written informed consent was obtained from each participant after a careful explanation of the concept and purpose of the study. The participants were ensured of privacy and confidentiality. The study protocol was reviewed and approved by the DMIMS(DU)/IEC/2019/8007.

## Results

Table 1 comprises the demographic characteristics of 40 participants, including age group, gender, presence of diabetes, duration of diabetes, and hypertension. The mean age of the participants was 61.48 years.

**Table 1:** Distribution of participants with characteristics

Age Group	Number of Patients	Percentage (%)
20-29 years	1	2.5%
50-59 years	8	20%
>60 years	31	77.5%
Gender		
Male	20	50%
Female	20	50%
Diabetes		
Known cases	28	70%
Newly diagnosed	12	30%
Duration of Diabetes		
1-5 years	11	39.29%
6-10 years	13	46.43%
11-15 years	4	14.29%
Hypertension		
Yes	22	55%
No	18	45%

Table 2 shows the visual acuity for patients in both eyes. The table presents the distribution of eyes based on visual acuity, categorizing them into two groups: normal (6/6 to 6/18) and less (<6/18).

**Table 2:** Distribution of eyes according to visual acuity

Visual Acuity	No. of eyes	Percentage
Normal (6/6 to 6/18)	23	28.75
Less (<6/18)	57	71.25
Total	80	100

Table 3 presents data on the occurrence of diabetic retinopathy and its different subtypes, drawn from a sample of patients. It offers information on the overall number of patients who underwent examination, the number of patients who exhibited diabetic retinopathy, and the corresponding percentage of those affected in the sample. Table 4, on the other hand, presents the breakdown of patients with diabetic retinopathy according to distinct age groups.

**Table 3:** Prevalence of diabetic retinopathy and subtypes NPDR: non-proliferative diabetic retinopathy, PDR: proliferative diabetic retinopathy, DMO: diabetic macular edema.

	Total Patients	Prevalence
Diabetic retinopathy	17	42.5%
NPDR	12	30%
PDR	0	0%
DMO	5	12.5%
Mild NPDR	5	12.5%
Moderate NPDR	7	17.5%
Severe NPDR	0	0%

**Table 4:** Age distribution of patients with diabetic retinopathy

Age Group	Number of Patients	Percentage
50-59 years	5	29.41%
>60 years	12	70.59%

Table 5 provides an overview of the gender distribution among patients diagnosed with diabetic retinopathy.

**Table 5:** Gender distribution among patients with diabetic retinopathy

Gender	Number of Patients	Prevalence
Male	8	47.06%
Female	9	52.94%

Table 6 provides information on the duration of diabetes mellitus among patients diagnosed with diabetic retinopathy. 

**Table 6:** Duration of diabetes mellitus among diabetic retinopathy patients

Duration of Diabetes	Number of Patients	Percentage
6-10 years	13	76.47%
11-15 years	4	23.53%
More than 15 years	0	0%

Table 7 presents information on ophthalmic conditions and hypertension among patients diagnosed with diabetic retinopathy. 

**Table 7:** Ophthalmic conditions and history of hypertension among diabetic retinopathy patients

Ophthalmic Conditions	Number of Patients	Percentage
Senile cataracts	11	64.71%
History of hypertension	13	76.47%

Table 8 presents information on patient awareness regarding diabetes mellitus and its association with diabetic retinopathy.

**Table 8:** Patient awareness about diabetes mellitus and diabetic retinopathy

Awareness Information	Yes	Percentage (%)	No	Percentage (%)
Knowledge about diabetes mellitus	28	70%	12	30%
Awareness of diabetes as an ophthalmic disease	3	7.5%	27	92.5

## Discussion

The present study assesses the occurrence of diabetic retinopathy in diabetic patients attending a tertiary care center in Central India. The study population showed 42.5% of patients had diabetic retinopathy, comparable to the recent studies in this zone. Preceding surveys in sizeable populations revealed lower percentages of diabetic retinopathy, such as 5.6% in rural China [4], 7.2% in parts of Turkey [5], 7.6% in Kuwait [6], and as high as 78% in Jamaica [7].

Recent studies in India revealed other data for the prevalence of diabetic retinopathy, such as 21.7% on a pan-India scale. The incidence was 12.27% in central India and 34.06% in North India, 15% in an overall study in India [8], 14.3% in Pune [9], and an incidence of 21.89% and progression of 33.45% [10] in the Singapore Indians in a study done for six years. Although direct comparisons among studies are challenging due to differing methodologies, areas or country or geographical differences taken for such studies, definitions used for diagnosis, and various population characteristics, these levels are, nevertheless, significant. The results were analogous to previous studies, and the higher prevalence of NPDR evaluated against PDR was akin to previous observations made in South India.

It was observed that out of the diabetic patients, most patients suffered from moderate NPDR (17.5%). Mild NPDR was seen in 12.5% of the total patients and diabetic maculopathy in 12.5%. Thus, out of the patients with diabetic retinopathy, 29.41% had DM, 29.41% had mild NPDR, and 41.18% had moderate NPDR. Most of the patients suffering from diabetic retinopathy were upward of 60 years of age, with lesser frequency in the 50- to 59-year age group.

The preponderance was slightly higher among females (52.94%) than among males (47.06%). Previous studies have shown varying results regarding the association of gender with diabetic retinopathy. In the Joslin Clinic patients, females had a greater onset of diabetic retinopathy in old age, but the number of males and females was equal in those with PDR [11]. Some other studies, like CURES Eye Study [12], the UKPDS study [13], and the Hyderabad Study [14], show the prevalence of males to be significantly higher than females.

The duration of diabetes mellitus has a noteworthy impact on the development of diabetic retinopathy, as none of the patients having a history of one to five years suffered from diabetic retinopathy, all 13 patients with a history of DM of six to 10 years, and all four patients with a history of DM of 11-15 years progressed to diabetic retinopathy. The reasons for this are the vascular and hematological changes that succeed in diabetes mellitus, such as endothelial cell damage, thickening of the capillary basement membrane, rouleaux formation by RBCs, and an increase in the adhesiveness of platelets. Changes in plasma viscosity promote microvascular occlusion and compromise retinal blood flow leading to ischemia. This ultimately leads to microaneurysms, retinal hemorrhages, and retinal edema, culminating in signs of DR. The most widespread and longest survey in Ophthalmology, the Wisconsin Epidemiologic Study of Diabetic Retinopathy (WESDR), reported that patients having diabetes for longer periods showed a greater incidence of diabetic retinopathy [15]. In Indian studies, too, the increased frequency of diabetic retinopathy has been related to longstanding diabetes [12]. ‘CURES’ has established that with every five-year increase in diabetes, the vulnerability to diabetic retinopathy is increased by 1.89 times [12].

Eleven (64.71%) patients with diabetic retinopathy also had senile cataracts. The Palakkad Eye Disease Survey stated that cataracts (27.8%) resulted in serious visual impairment in diabetic patients [16]. In another study, of diabetic patients, who underwent cataract surgery, 44% of patients showed progression of diabetic retinopathy and 8% developed diabetic retinopathy for the first time [17]. Removal of a cataract may aggravate existing diabetic maculopathy as well as NPDR. Thus, before cataract extraction, routine retinal documentation and detection of diabetic retinopathy are imperative [18-20].

Thirteen (76.47%) patients had a history of hypertension; retinal capillary endothelial cells in diabetic patients are damaged due to high blood pressure that creates an excess force of blood flow. Hypertension can lead to diabetic retinopathy due to impaired autoregulation and hyperperfusion and release of proangiogenic vascular endothelial growth factor (VEGF) [21-25]. The UKPDS showed a relationship between a higher incidence of diabetic retinopathy and systolic blood pressure [13]. In contrast, the WEDSR showed a significant relationship between the progression of diabetic retinopathy and diastolic blood pressure [15]. Thirty percent of the total cases were newly diagnosed cases of diabetes mellitus. This greatly impacts the Public Health Awareness Programme, signifying the necessity for regular eye examinations of diabetics for screening diabetic retinopathy patients [26].

The high prevalence necessitates the creation of awareness among the adult population, especially those with deranged blood glucose levels and comorbidities like cataracts and hypertension. It is paramount to sensitize the public about the detrimental effects of diabetic retinopathy [27,28]. This can be done by increasing visibility about the consequences of DR through posters and flyers in hospitals and primary healthcare centers and social media. As this condition is a major cause of vision loss in the working age group, organizing talks and seminars in the workplace will give them first-hand knowledge and educate their families. Collaborations with non-governmental organizations (NGOs) and global health programs will contribute substantially to spreading awareness among all segments of society [29-31]. Taking advantage of modalities like telemedicine to contact specialty doctors for routine monitoring and regular follow-up can be an efficient tool in the screening of a multitude of diabetic patients. There are several programs in India, like ICMR-AROGYASREE, NeHA, and VRCs, that can help bridge the gap between specialized doctors in urban areas and rural patients [32].

## Conclusions

This study draws attention to the higher frequency of diabetic retinopathy among diabetic patients in central India. About 42.5% of the patients showed positive findings for DR. The prevalence is significantly higher in old age. The study also shows an association with factors such as a long history of diabetes, hypertension, cataract, high blood sugar levels, and unilateral or bilateral low vision. The high numbers of newly diagnosed cases of diabetes mellitus suggest the importance of screening patients in Ophthalmology OPD for swift intervention and rehabilitation. The study also created awareness among the diabetes mellitus and diabetic retinopathy patients who were examined for the duration of the study. This study had some limitations. Prevalence may not depict the true picture of diabetic retinopathy in the areas due to hidden cases, i.e., undiagnosed cases. The required sample size is small and further community-based studies on a finding of the prevalence of diabetic retinopathy are required. We are unable to conduct other required investigations like urine routine and microscopy, lipid profile, liver function test (LFT), and kidney function test (KFT) which would have given us better and more precise findings and results in association with diabetic retinopathy. We recommend that further larger studies are required to evaluate the ascendancy of diabetic retinopathy in diabetic patients in India. Uncontrolled PDR can ultimately progress to advanced diabetic eye disease that encompasses vitreous hemorrhage, tractional retinal detachment, and neovascular glaucoma. This validates the need for metabolic control of diabetes and early screening and investigation of diabetic patients so that diabetic retinopathy can be diagnosed and managed in the initial stages.
